# Using Pedigree and Genomic Data toward Better Management of Inbreeding in Italian Dairy Sheep and Goat Breeds

**DOI:** 10.3390/ani12202828

**Published:** 2022-10-18

**Authors:** Matteo Cortellari, Alessio Negro, Arianna Bionda, Silverio Grande, Alberto Cesarani, Antonello Carta, Nicola Macciotta, Stefano Biffani, Paola Crepaldi

**Affiliations:** 1 Dipartimento di Scienze Agrarie e Ambientali—Produzione, Territorio, Agroenergia, Università degli Studi di Milano, 20133 Milan, Italy; 2 Ufficio Studi, Associazione Nazionale della Pastorizia (Asso.Na.Pa.), 00187 Rome, Italy; 3 Dipartimento di Agraria, Università degli Studi di Sassari, 07100 Sassari, Italy; 4 Department of Animal and Dairy Science, University of Georgia, Athens, GA 30602, USA; 5 Unità di Ricerca di Genetica e Biotecnologie, Agris Sardegna, 07100 Sassari, Italy; 6 Istituto di Biologia e Biotecnologia Agraria (IBBA), Consiglio Nazionale delle Ricerche, 20133 Milano, Italy

**Keywords:** small ruminant, inbreeding, SNP, pedigree, genomic inbreeding, runs of homozygosis, F_ROH_

## Abstract

**Simple Summary:**

The inbreeding coefficient is relevant for managing livestock and safeguarding biodiversity, especially in small populations. Small ruminant breeders mainly rely on pedigree information, but genomics is increasingly gaining ground as a tool to face possible pedigree inaccuracies. This study investigates pedigree-based (F_PED_) and genomic (F_ROH_ and F_GRM_) inbreeding in a representative number of Italian sheep and goat populations. Indeed, even though it has been widely studied in cattle, there is still little knowledge about the relationship between these coefficients in small ruminants, which are characterized by a different population structure, often with unconnected farms. Mean inbreeding values were low, F_ROH_ being the highest, with breed differences due to different managements; the correlation between F_PED_ and F_ROH_ was the strongest and directly related to pedigree depth. Moreover, we estimated F_PED_ from F_ROH_ using a linear regression model. Since massive genotyping is not affordable to small ruminant breeders, it is important to understand the distinction and relationship between differently calculated inbreeding coefficients, also in view of the introduction of genomic enhanced breeding values. Our study highlights the importance of accurate pedigree information and, especially if not obtainable, of genotyping animals. Correct data contribute to mitigate inbreeding depression and loss of genetic variability.

**Abstract:**

The inbreeding coefficient is an important parameter for livestock management. Small ruminant breeders and associations mainly rely on pedigree information, but genomic tools are gaining relevance, overcoming possible pedigree inconsistencies. This study investigates the relationship between pedigree-based and genomic inbreeding in two goat and four sheep dairy breeds. Pedigree and genomic data (medium-density SNPchip) were obtained for 3107 goats and 2511 sheep. We estimated pedigree depth (number of fully traced generations, FullGen) and inbreeding (F_PED_), as well as two genomic inbreeding indexes, using runs of the homozygosity (F_ROH_) and genomic relationship matrix (F_GRM_). The correlation between the inbreeding coefficients was assessed. A linear regression model (LRM) was fitted for estimating F_PED_ from F_ROH_. After quality control on genomic data, we retained 5085 animals. Mean inbreeding values were low, with higher F_ROH_ than F_PED_ and F_GRM_. Breed differences can partially depend on different managements. The correlation between F_PED_ and F_ROH_ was the highest and directly related to pedigree depth. The best LRM was chosen for FullGen ≥4 and ≥6 for goats and sheep, respectively; after excluding animals with extreme residuals, a new refined regression equation was calculated. Since massive genotyping is not affordable to small ruminant breeders, it is important to understand the distinction and relationship between differently calculated inbreeding coefficients, also in view of the introduction of genomic enhanced breeding values. Our study highlights the importance of accurate pedigree information and, especially if not obtainable, of calculating genomic-based inbreeding coefficients. A better estimation of animals’ relatedness contributes to improve animal breeding and conservation.

## 1. Introduction

The accurate estimate of the inbreeding value of an individual could be of extreme importance for all the domesticated species, both for commercial and conservation purposes [1,2,3]. With regards to small ruminants, pedigree information continues to be the most widely used tool to compute inbreeding coefficients, even though several authors have pointed out that pedigree-based inbreeding values can be severely underestimated due to the high level of inconsistencies in these species [4,5]. Indeed, in goats and sheep, the correct parentage can be difficult to assess due to several factors, such as the high missing information rate, the limited use of artificial insemination, the creation of mating groups with the simultaneous presence of more males [6] and the extensive farming system [7]. According to Legarra et al. (2014) [8], the unknown fatherhood rate reaches 50% and 20% in Latxa and Manech/Basco-Béarnaise sheep breeds, respectively.

Today, genomic data obtained by high-throughput SNP genotyping represent a new instrument that should be integrated with the traditional pedigree information to more precisely estimate the relationship between individuals [9]. Moreover, genomic information allows for a more accurate inbreeding estimation by overcoming pedigree inconsistencies and by accounting for the Mendelian sampling. This allows to better decipher the genetic variation between the individuals, which is of great interest primarily in small and endangered populations, and to eventually estimate unbiased genetic values [10,11].

There are different ways to calculate inbreeding using SNP chip data, and those based on runs of homozygosity (ROH) and the genomic relationship matrix (GRM) are among the most used [12,13]. ROH are segments of homozygous genotypes identified for a single individual and composed of a series of identical haplotypes. ROH provide information about a subject’s level of autozygosity, age of the inbreeding events and origin, but most importantly, they can be used to assess a reliable inbreeding coefficient (F_ROH_) [4]. Indeed, recombination events can disrupt long stretches of DNA over generations (or meiosis), allowing to estimate the age of the inbreeding events: Short ROH derive from more distant ancestors, whereas long ROH suggest that the autozygosity of an individual comes from more recent meiosis events, leading to the presence of a recent common ancestor [14].

Even if SNP genotyping has been routinely implemented in dairy cattle [15], its costs are still too high compared to the value of a single sheep or goat; thus, small ruminant breeders and breeder associations cannot afford to genotype a large number of animals. This supports the need to find a way to integrate traditional pedigree data with the genomic data and eventually estimate reliable inbreeding coefficients. Some previous studies have already investigated the genomic variability of Italian sheep and goat breeds [16,17,18,19]. However, their results were based on a limited number of animals and/or did not elucidate the relationship between traditional pedigree-based and innovative genomic-based parameters. For this reason, our work studies the relationship between F_PED_ and pedigree depth and the genomic inbreeding F_ROH_ and F_GRM_ in six Italian small ruminant populations focusing, in particular, on F_PED_ and F_ROH_.

## 2. Materials and Methods

### 2.1. Breeds

For this work, we selected a total of six breeds, namely four local dairy sheep breeds—Comisana, Delle Langhe, Massese and Sarda—and two cosmopolite dairy goat breeds—Camosciata delle Alpi and Saanen. All the breeds are mainly used for cheese production, being both representative of different production systems and geographical areas. The official herd books of these breeds are managed by the Italian Sheep and Goat Breeders Association (Rome, Italy), hereinafter referred to as Asso.Na.Pa. In all the breeds, male selection candidates are DNA-tested for parentage assignment. Artificial insemination (AI) is used mainly in Camosciata delle Alpi and Saanen breeds, where from 10 to 15% of annual births are from semen of foreign AI bucks. All breeds are officially milk-recorded, and a routine genetic evaluation for milk yields is in place. The selection scheme for Comisana and Massese sheep breeds is based on two different closed nucleus flocks reared at the Genetic Center of Asciano (Siena, Italy); instead, the Sarda sheep is managed using a pyramidal scheme, with a nucleus flock at the apex [20]. Finally, selection in Delle Langhe sheep aims to improve milk production through the estimate of the breeding value for milk yield (expressed in kg); a software is available for breeders to optimize genetic breeding values and manage mating to reduce offspring inbreeding.

### 2.2. Datasets and Quality Control

Pedigree records and genomic data were obtained from the ‘Conservation, Health and Efficiency Empowerment of Small Ruminant’ (CHEESR) repository project, a database created by Asso.Na.Pa. in the framework of the Italian National Rural Development Plan (PSRN)—sub-measure 10.2 project and supported by the European Agricultural Fund for Rural Development (EAFRD). In total, we analyzed 3107 goats (2164 Camosciata delle Alpi and 943 Saanen) and 2511 sheep (1498 Sarda, 534 Comisana, 375 Massese and 104 Delle Langhe). All animals were genotyped with the medium density chip, Ovine SNP50 BeadChip for ewes and Goat SNP50 BeadChip for goats. A quality control analysis was performed on both species fitting the same parameters and using PLINK 1.9 software [21]: A missing genotype frequency ≥ 0.05 and an individual call rate below 0.95 were the thresholds to exclude SNP or animals, respectively. In addition, all the unplaced markers (according to the assembly versions ARS1 for goat and OAR4 for sheep) and those located on the X chromosome were excluded in accordance with the majority of the Literature and given the small number of SNPs (about 3% of the total).

### 2.3. Pedigree Analysis

All the pedigree analyses were carried out using the *optiSel* package of the R software [22]. The function ‘summary.Pedig’ was used to calculate the pedigree-based inbreeding (F_PED_), the number of fully traced generations (i.e., whose ancestors are all known, FullGen), the number of maximum generation (MaxGen), the number of equivalent complete generations (EquiGen), the percentage of animals with a complete first generation and the effective size for each sheep or goat breed according to Wright’s formula [23].

### 2.4. Genomic Inbreeding Calculation

We used the function ‘--homozyg’ of PLINK 1.9 for the calculation of the ROH applying the following criteria: (i) a sliding window of 20 SNPs, (ii) no heterozygous genotype allowed, (iii) no more than two missing genotypes, (iv) a minimum number of SNPs in a ROH equal to 20, (v) a minimum ROH length of 1 Mb, (vi) a minimum SNP density of 1 SNP per 500 kb and (vii) a maximum gap of 500 kb between two consecutive homozygous SNPs. In order to minimize false positives discovery within regions of low marker density, rather stringent criteria were selected. ROH distribution was characterized for each breed by estimating the number of individuals without ROH, the mean number of ROH per individual, the mean total length of ROH per individual, the mean length of a ROH per individual and the genomic inbreeding coefficient (F_ROH_) in each individual. The F_ROH_ coefficients were computed using the following equation:

(1) $$F_{ROH,i}=\frac{L_{ROH,i}}{L_{AUTO}}$$

where L_ROH_ is the sum of the total length of ROH in individual *i* and L_AUTO_ is the total length of the autosomes covered by the SNPs [24]. In addition, we categorized all the estimated ROH in five different ROH length classes: from 1 to 2 Mb, from 2 to 4 Mb, from 4 to 8 Mb, from 8 to 16 Mb and over 16 Mb. ROH length can represent the starting point for the evaluation of how many generations ago an inbreeding event occurred: Indeed, recent ROH segments are longer because of the less probability of being interrupted by recombination events; on the other hand, more ancient ones are shorter. In particular, F_ROH_ are expected to correspond to the ancestral population dating 50 (1 < ROH < 2), 20 (2 < ROH < 4), 12.5 (4 < ROH < 8), 6 (8 < ROH < 16) and 3 (ROH > 16) generations ago [25].

The computation of the GRM and the consequent F_GRM_ values was performed through the use of the ‘--ibc’ parameter implemented in the GCTA v1.93.3 software [26]. Whereas no minor allele frequency (MAF) threshold was applied to allow a better estimation of ROH [27], before the estimation of the F_GRM_ we excluded the SNPs with a MAF < 0.05, as this parameter is strongly influenced by the frequencies of rare alleles [28].

### 2.5. F_PED_-F_ROH_ Estimate

Using the functions ‘cor’ and ‘lm’ implemented in the R base package, we calculated the correlation coefficient between F_ROH_, F_GRM_ and F_PED_, and we fitted a linear model to estimate the most probable F_PED_ value from genomic data (F_ROH_). This calculation was performed on all the individuals of both species together considering increasing FullGen classes (i.e., increasing number of known ancestors).

Then, for each species, we chose the linear model of the FullGen class that maintained at least 600 individuals of the species of interest and, at the same time, had both the highest correlation coefficient between F_PED_ and F_ROH_ and the highest linear model R^2^. After that, we applied the coefficients of the chosen FullGen model to estimate the F_PED_ value only on the individuals of the correspondent species with the selected minimum FullGen. We calculated the standardized residuals (Z-score of the differences between the predicted and observed F_PED_ values) and excluded all the animals with a standardized residual falling under the 1st or over the 3rd quartile. Lastly, we recalculated the linear model using only the retained subjects and re-estimated their F_PED_ values with the coefficient of this last refined model. 

## 3. Results

### 3.1. Dataset Creation

We applied the same thresholds and filtering parameters to both sheep and goat datasets. After the quality control, we retained 3086 (2147 Camosciata delle Alpi and 939 Saanen) individuals and 51,097 markers for goats, and 2484 (1480 Sarda, 529 Comisana, 371 Massese and 104 Delle Langhe) individuals and 46,723 markers for sheep. All the 485 individuals for which we could not calculate F_GRM_, F_ROH_ or F_PED_ were excluded. Therefore, the final dataset used for estimating the correlation coefficients and the linear model included 5085 animals (3028 goats and 2057 sheep) (Table 1). The results of pedigree structure of each of the six breeds considered are reported in Supplementary Table S1.

### 3.2. Inbreeding Correlation and Linear Model

**Figure 1:**
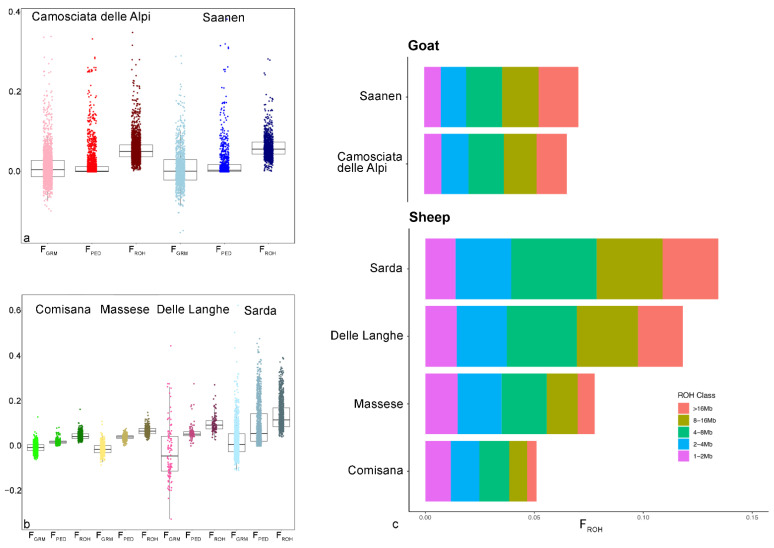
Distribution of F_GRM_, F_PED_ and F_ROH_ in goat (**a**) and sheep (**b**) breeds, and F_ROH_ per ROH length class in the examined breeds (**c**).

For each sheep and goat breed, we calculated F_PED_, F_GRM_ and F_ROH_ for different ROH length classes. An F_PED_ value equal to 0 was observed in less than 1% of sheep and 37% of goats. As shown in Figure 1a,b, the mean F_ROH_ values were higher than the other inbreeding coefficients in both the species. Instead, F_PED_ values were higher than F_GRM_ in sheep, whereas the two were similar in goats. It is worth noticing that F_GRM_ showed some negative values in all the breeds and a particularly spread distribution in the Delle Langhe breed. 

Regarding the F_ROH_ distribution per ROH length class (Figure 1c), the two goat breeds had approximately the same distribution for all the F_ROH_ classes apart from the one derived from ROH > 16 Mb (suggesting recent inbreeding), which was higher in Saanen than in Camosciata delle Alpi. In the sheep species, Sarda and Delle Langhe breeds presented the highest F_ROH_ value and proportion of long ROH classes. In any case, all inbreeding coefficients were relatively low in both species, with the highest values in the Sarda sheep.

The correlation coefficients between F_GRM_, F_ROH_ and F_PED_ were much higher in sheep than in goats, and, in particular, in the Sarda breed. In addition, the correlation between F_PED_ and F_ROH_ was higher than between F_PED_ and F_GRM_; therefore, we decided to use a linear regression model to estimate F_PED_ from F_ROH_ (Table 1).

The correlation coefficients between F_PED_ and F_ROH_ and the R^2^ of the linear model, reported in Table 2, showed a progressive increment as the minimum FullGen increased until we reached FullGen > 6. All the correlations were statistically significant (*p* < 0.0001). Based on these results and the assumptions explained in the material and methods section (highest correlation value and R^2^, and at least 600 subjects), we identified the model calculated for FullGen ≥ 4 as the best one for the goats, and the one calculated on Fullgen ≥ 6 as the best for the sheep.

#### 3.2.1. Goats

**Figure 2:**
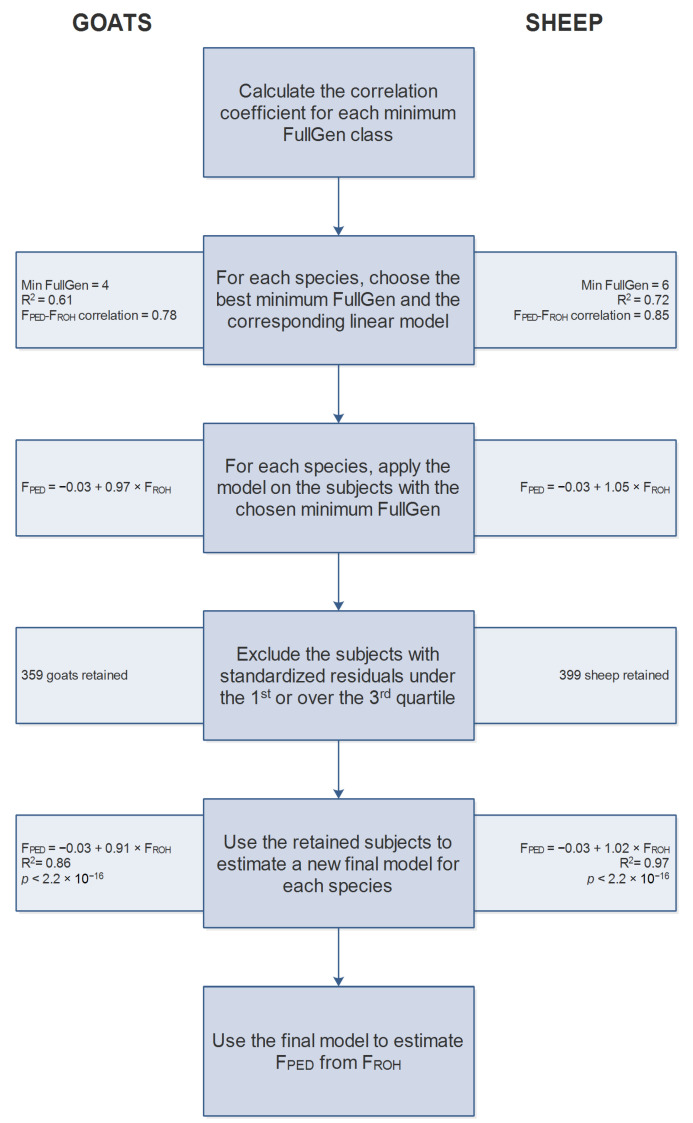
Flowchart of the estimation of a linear regression model estimating F_PED_ from F_ROH_ for goats and sheep.

**Figure 3:**
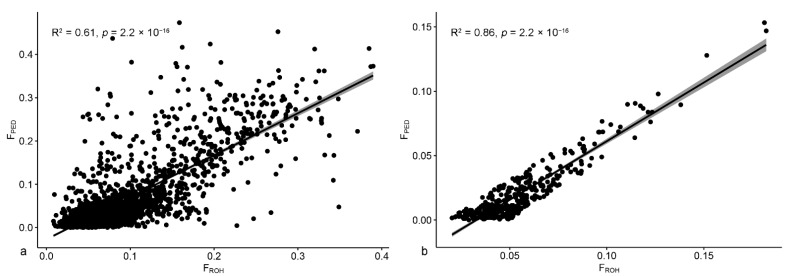
Linear regression models to estimate F_PED_ from F_ROH_. (**a**) Linear regression model calculated on all the animals with FullGen ≥ 4. (**b**) Refined model (removing animals falling below the 1st and above the 3rd quartile of the standardized residual distribution) for goats with FullGen ≥ 4.

Following the method shown in Figure 2, we applied the coefficients of the linear model calculated on all the animals with FullGen ≥ 4 (intercept = −0.03, slope = 0.97) on the 717 goats with FullGen ≥ 4 to estimate F_PED_ values (Figure 3a). Then, we excluded the 358 goats with a standardized residual falling under the first and over the third quartiles, and we calculated the new regression equation on the remaining goats: F_PED_ = −0.03 + 0.91 × F_ROH_ (R^2^ = 0.86, *p* < 2.2 × 10^−16^) (Figure 3b).

Lastly, we used this last equation to estimate the definitive F_PED_ from F_ROH_ for all the goats with FullGen ≥ 4, and for each 0.05 class of estimated definitive F_PED_, we calculated the corresponding mean and range of F_ROH_ (Table 3). All data are reported, but due to the low number of subjects in some of the estimated F_PED_ classes, only those with a relevant size are further commented. For example, it should be noted that for the class of estimated definitive F_PED_ 0–0.05, the corresponding F_ROH_ values were higher in Saanen than in Camosciata delle Alpi.

#### 3.2.2. Sheep

**Figure 4:**
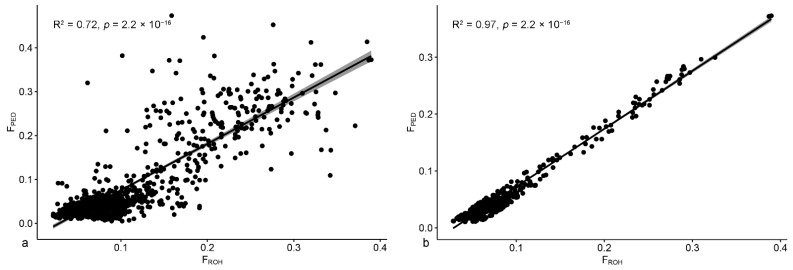
Linear regression models to estimate F_PED_ from F_ROH_. (**a**) Linear regression model calculated on all the animals with FullGen ≥ 6. (**b**) Refined model (removing animals falling below the 1st and above the 3rd quartile of the standardised residual distribution) for sheep with FullGen ≥ 6.

For sheep, we selected the coefficients relative to the regression model calculated for animals with a minimum FullGen equal to 6 (intercept = −0.03, slope = 1.06) and applied them on the 927 sheep with a FullGen ≥ 6 to calculate the estimated F_PED_ values (Figure 4a). We standardized the residuals and excluded 463 subjects falling under the first and over the third quartiles. Finally, we calculated the new equation using the 464 retained sheep: F_PED_ = −0.03 + 1.02 × F_ROH_ (R^2^ = 0.97, *p* < 2.2 × 10^−16^) (Figure 4b).

The estimated F_PED_ intervals and relative F_ROH_ means and ranges for all the sheep with FullGen ≥ 6 are reported in Table 4. There was a significant difference in the breeds’ mean F_ROH_ corresponding to an estimated F_PED_ of 0.00–0.05 and 0.05–0.10: Comisana had the lowest F_ROH_ values, followed by Massese, while Delle Langhe and Sarda present the highest values. As shown in Table 4, only Sarda animals were found in the higher F_PED_ classes (>0.20).

## 4. Discussion

The inbreeding coefficient is a fundamental tool for livestock husbandry: It is necessary for increasing the accuracy of the genomic and genetic breeding value of animals and the consequent improving of the breeds [29]; it is one of the main parameters to monitor the selection programs; it is also useful in biodiversity conservation, allowing to identify possible pitfalls in terms of loss of genetic variability; and it helps breeders to choose the best mating schemes to improve animal welfare and to avoid inbreeding depression (which is especially relevant when using the AI) [30]. As previously mentioned, genomic inbreeding coefficients are more reliable than pedigree-based ones, being free from possible registration inaccuracy [4,11]. This is the reason why the implementation of genomic tools at the field level has increased, leading to genomic selection programs not only in cattle but also in other farm animals such as sheep and goats [31,32,33].

Nevertheless, the genotyping costs, although consistently reduced in recent years, cannot yet be afforded by the breeders of small ruminants. This makes it necessary to understand how to correctly interpret and compare inbreeding coefficients calculated from different data. Therefore, the present study aimed to explore the F_PED_ and genomic inbreeding coefficients (F_GRM_ and F_ROH_), as well as their relationship, in a representative number of animals belonging to six differently managed Italian dairy small ruminant breeds.

Our results showed that there were some differences in the variability distribution of inbreeding coefficients, both within and among breeds. As we know from the literature, F_GRM_ values tend to be more variable with respect to the other inbreeding parameters because they are highly influenced by the rare allele variants frequencies [12]; moreover, the particularly spread distribution found in the Delle Langhe breed could also depend on its smaller sample compared to the other breeds. Regarding the wide range of F_PED_ values identified by the analysis, we know that goat pedigrees are less complete compared to other species, similar to a lot of animals (almost 40%) with F_PED_ = 0, often due to lack of genealogic information. For the same reason, the correlation between F_ROH_ and F_PED_ coefficients is lower in goats. Regarding the percentage of F_ROH_ per ROH length class, the different management of the breeds can account for a portion of the observed variability: For example, the higher level of recent inbreeding found in Saanen compared with Camosciata delle Alpi could partially depend on the higher use of AI in the former (33% vs. 25% in our samples, higher than the values, 11% and 12%, estimated in the whole populations enrolled to the herd books in the same years). The inbreeding estimated on these subjects was, for the most part, low. The lack of very inbred animals could be considered a limitation of the present study, but it should also be considered that this can depend either on good management of the population or from the low level of connection of the Italian small ruminant farms, with a minimal exchange of animals except when the breeders make use of the AI or receive animals from a genetic center. The diffusion of AI is still limited in both species; however, it is rising in goats due to the commercialization of foreign, mainly French, semen. Avoiding an excessive increase of inbreeding is a main goal of the breeders and their associations to prevent inbreeding depression, especially in small-sized populations, whose survival would be in danger if a reduction of the number of the breeding animals occurred. If the genomic inbreeding could be estimated for all the animals of a farm, or at least a representative number, it would be possible to warn the breeders who are at risk of inbreeding-related issues.

The comparison of inbreeding coefficients with those reported in literature is hampered by the several different parameters that can be defined to estimate them. The average F_PED_ in a population varies based on the considered pedigree depth (i.e., number of generations tracked back), the use of unknown parent groups and the involved computational algorithm [34], some of which can estimate non-zero inbreeding coefficients also for animals with unknown parents [35]. F_ROH_ can be estimated starting from sliding (as in the present study) or consecutive (not overlapping regions) ROH, both varying according to the parameters used to define the regions. In the literature, results about this parameter have also considerably changed within the same breed. One example is the Sarda dairy sheep: In this study, the average F_ROH_ for this breed was 0.135 using sliding regions, whereas Cesarani et al. (2019) [32] reported a value of 0.059 using consecutive regions with more strict parameters and Mastrangelo et al. (2018) [18] estimated an average F_ROH_ of 0.041. In the latter study, the authors reported F_ROH_ values also for Delle Langhe (0.080 vs. 0.099 in this study), Comisana (0.016 vs. 0.044 in this study) and Massese (0.055 vs. 0.067 in this study). The different values between Cesarani et al. (2019) [18] and the present study are mainly due to the different sample size and the minimum number of SNP to call a ROH, which was 30 in the previous study and 20 in the present one. Finally, F_GRM_ values change according to the method used to create the genomic relationship matrix [28,36,37] in which different scaling and weighting factors are used, and according to the quality control made on SNPs data, in particular allele frequency. In fact, some studies have suggested the use of fixed allele frequency (0.5) when building the GRM. According to Bjelland et al. (2013) [38], this kind of matrix is a homozygosity measure adjusted to conform to the distribution of the pedigree inbreeding and, therefore, the correlations between F_PED_ and F_GRM_ estimated with this fixed frequency are extremely high.

The analysis of the relationship among inbreeding coefficients showed that the correlation of F_PED_ was generally higher with F_ROH_ (0.30 in goats and 0.82 in sheep) than F_GRM_ (0.27 and 0.35, respectively), especially when the pedigrees were more complete. The results of our analyses are consistent with those previously reported in other studies [12,39]. Of interest is the negative correlation observed between F_PED_ and F_GRM_ in Comisana and Massese breeds and that already reported in the literature for other species [40]. Pedigree depth was directly related to the correlation between F_ROH_ and F_PED_; this means that in order to estimate F_PED_ accurately, between four and six full generations should be known. It would be important to bring this information to the attention of the breeders, who should especially genotype those animals with a small number of known ancestors. We used a simple regression model that aims to estimate a more accurate pedigree-based inbreeding coefficient on the basis of F_ROH_ and can be useful for the breeders to compare the inbreeding of genotyped and non-genotyped animals. This regression model could obviously be improved using a larger sample size per breed and including highly related animals. We noticed that goats had shallower pedigrees and a higher number of inbreeding coefficients equal to zero. For this reason, since the two species share several characteristics, such as similar management and production aptitude (milk and cheese) and they are often raised together, the initial model included both species. The results of the model also highlighted that different breeds had different mean F_ROH_ per estimated F_PED_ class. This aspect should be considered when implementing the genomic inbreeding in the animal evaluation, for example, through the estimation of genetic breeding values. Indeed, previously published works have attempted to propose a new model based on ROH for the computation of genomic breeding values in cattle (G_ROH_), finding quite high levels of accuracy [41]. This could be a very useful solution in small ruminant populations in which we have less accurate pedigree information and a lower level of connection between farms.

## 5. Conclusions

Pedigree information is the most used source of data to compute inbreeding coefficients, especially in small ruminants, due to the still high cost of genotyping. As emerged from our analyses on Italian sheep and goat populations, when all data were included, there were low values of correlation between inbreeding calculated from pedigree F_PED_ and genomic data F_ROH_, particularly in goats (0.30). Indeed, F_PED_ values equal to zero (almost 40% in goats) occur often due to a lack of genealogic information. Excluding subjects with likely erroneous or too shallow pedigrees while keeping an adequate sample size allowed us to better estimate a reliable correlation between the two inbreeding coefficients. These results underline the importance for small ruminant breeders and associations to improve the accuracy of the collection of pedigree information and, especially when it is not possible, the significant advantages of estimating a genomic inbreeding coefficient. The correctness of the data, indeed, allows to improve small ruminant breeds in different ways: having more reliable EBV and creating better mating schemes in order to improve welfare, production and health-related traits; minimizing the risk of inbreeding depression; and better understanding the effect of their management on inbreeding trends. On the contrary, inaccurate information might limit the breed’s genetic progress and lead to the reduction of the genetic variability, which is essential to safeguard livestock biodiversity and to face future climate challenges.

## Figures and Tables

**Table 1:** Number of subjects in the final dataset for each species and breed, relative mean value of pedigree, ROH and GRM-based inbreeding coefficients (F_PED_, F_ROH_ and F_GRM_) and their correlation coefficients.

Species/Breed	N. Subjects	Mean F_PED_	Mean F_ROH_	Mean F_GRM_	F_PED_-F_ROH_ Correlation Coefficient (*p*-Value)	F_PED_-F_GRM_ Correlation Coefficient (*p*-Value)	F_ROH_-F_GRM_ Correlation Coefficient (*p*-Value)
**Goats**	3028	0.017 ± 0.039	0.059 ± 0.032	0.012 ± 0.046	0.278 (<0.0001)	0.272 (<0.0001)	0.604 (<0.0001)
Camosciata delle Alpi	2093	0.016 ± 0.038	0.057 ± 0.033	0.013 ± 0.043	0.260 (<0.0001)	0.228 (<0.0001)	0.671 (<0.0001)
Saanen	935	0.020 ± 0.042	0.062 ± 0.030	0.010 ± 0.051	0.312 (<0.0001)	0.351 (<0.0001)	0.492 (<0.0001)
**Sheep**	2057	0.062 ± 0.076	0.097 ± 0.063	0.006 ± 0.064	0.817 (<0.0001)	0.365 (<0.0001)	0.477 (<0.0001)
Sarda	1053	0.093 ± 0.095	0.135 ± 0.065	0.023 ± 0.073	0.804 (<0.0001)	0.346 (<0.0001)	0.489 (<0.0001)
Delle Langhe	104	0.060 ± 0.037	0.099 ± 0.038	−0.025 ± 0.127	0.436 (<0.0001)	0.225 (0.022)	0.354 (<0.0001)
Comisana	529	0.018 ± 0.010	0.044 ± 0.016	−0.008 ± 0.021	0.379 (<0.0001)	−0.130 (0.003)	0.229 (<0.0001)
Massese	371	0.039 ± 0.010	0.067 ± 0.017	−0.013 ± 0.025	0.318 (<0.0001)	−0.070 (0.181)	0.224 (<0.0001)

**Table 2:** Number of goats and sheep, F_ROH_-F_PED_ correlation coefficients and *p*-value, and linear regression model (LRM) coefficients and R^2^ per minimum class of fully traced generations in the pedigree (FullGen).

Minimum FullGen	N. Animals	N. Goats	N. Sheep	Correlation Coefficient	LRM Intercept	LRM Slope	LRM R^2^
0	5085	3028	2057	0.712	−0.028	0.860	0.507
1	4549	2493	2056	0.725	−0.028	0.888	0.526
2	3911	1877	2034	0.735	−0.027	0.910	0.540
3	3311	1358	1953	0.753	−0.026	0.937	0.567
4	2522	717	1805	0.782	−0.027	0.971	0.611
5	1602	167	1435	0.825	−0.028	1.010	0.681
6	927	18	909	0.849	−0.030	1.056	0.720
7	378	2	376	0.847	−0.029	1.085	0.718
8	107	0	107	0.773	0.008	0.997	0.597

Correlation was significant (*p*-Value < 0.0001) for all the classes of FullGen.

**Table 3:** Number of goats, F_ROH_ range, mean, standard deviation (SD) and 95% confidence interval (CI) per each class of estimated F_PED_.

Breed	Estimated F_PED_ Class	N. Subjects	F_ROH_ Mean	F_ROH_ SD	F_ROH_ 95% CI	F_ROH_ Range
**Goats**	0.00–0.05	613	0.054	0.017	0.052–0.055	0.008–0.088
Camosciata delle Alpi	0.00–0.05	453	0.052	0.017	0.050–0.053	0.008–0.088
Saanen	0.00–0.05	160	0.058	0.015	0.056–0.061	0.024–0.088
**Goats**	0.05–0.10	81	0.103	0.013	0.101–0.106	0.088–0.138
Camosciata delle Alpi	0.05–0.10	49	0.102	0.011	0.099–0.105	0.089–0.132
Saanen	0.05–0.10	32	0.106	0.015	0.100–0.111	0.088–0.138
**Goats**	0.10–0.15	17	0.162	0.014	0.156–0.169	0.143–0.182
Camosciata delle Alpi	0.10–0.15	9	0.157	0.014	0.148–0.166	0.143–0.179
Saanen	0.10–0.15	8	0.168	0.012	0.159–0.176	0.153–0.182

**Table 4:** Number of sheep, F_ROH_ range, mean, standard deviation (SD) and 95% confidence interval (CI) per each class of estimated F_PED_.

Breed	Estimated F_PED_ Class	N. Subjects	F_ROH_ Mean	F_ROH_ SD	F_ROH_ 95% CI	F_ROH_ Range
**Sheep**	0.00–0.05	419	0.055	0.014	0.054–0.057	0.022–0.078
Comisana	0.00–0.05	163	0.045	0.013	0.043–0.047	0.022–0.078
Massese	0.00–0.05	198	0.061	0.010	0.059–0.062	0.029–0.078
Sarda	0.00–0.05	33	0.068	0.009	0.065–0.071	0.041–0.078
Delle Langhe	0.00–0.05	25	0.067	0.012	0.062–0.072	0.027–0.078
**Sheep**	0.05–0.10	231	0.097	0.014	0.095–0.098	0.078–0.127
Comisana	0.05–0.10	2	0.092	0.014	0.072–0.111	0.082–0.101
Massese	0.05–0.10	59	0.090	0.011	0.087–0.093	0.078–0.120
Sarda	0.05–0.10	122	0.099	0.015	0.096–0.101	0.078–0.127
Delle Langhe	0.05–0.10	48	0.100	0.013	0.096–0.103	0.079–0.124
**Sheep**	0.10–0.15	89	0.150	0.015	0.146–0.153	0.128–0.176
Comisana	0.10–0.15	1	0.162			
Sarda	0.10–0.15	79	0.149	0.014	0.146–0.152	0.128–0.176
Delle Langhe	0.10–0.15	9	0.152	0.018	0.140–0.164	0.128–0.175
**Sheep**	0.15–0.20	71	0.199	0.013	0.196–0.203	0.177–0.225
Sarda	0.15–0.20	68	0.199	0.013	0.196–0.203	0.177–0.225
Delle Langhe	0.15–0.20	3	0.203	0.022	0.178–0.227	0.179–0.222
**Sheep** (Sarda)	0.20–0.25	56	0.250	0.015	0.246–0.254	0.225–0.274
**Sheep** (Sarda)	0.25–0.30	27	0.293	0.014	0.288–0.298	0.275–0.321
**Sheep** (Sarda)	0.30–0.35	13	0.336	0.013	0.329–0.343	0.324–0.371

Only Sarda sheep had estimated F_PED_ values > 0.20.

## Data Availability

The data presented in this study are not publicly available and are part of the official herd book information managed by the Associazione Nazionale della Pastorizia (Asso.Na.Pa.). For further information, please contact S.G. (direzione@assonapa.it).

## Supplementary Materials

The following supporting information can be downloaded at: https://www.mdpi.com/article/10.3390/ani12202828/s1. Table S1: Breed pedigree information: maximum number of fully (FullGen), total (MaxGen), and equivalent complete (EquiGen) traced generations, % of subjects with a complete first generation and with MaxGen equal to 1, and effective population size (N_e_).
