# Supplementary material for: Using Pedigree and Genomic Data toward Better Management of Inbreeding in Italian Dairy Sheep and Goat Breeds
Source: Animals (Basel). 2022 Oct 18;12(20):2828. doi: 10.3390/ani12202828 (PMC9597836; doi:10.3390/ani12202828)
Supplement: Supplementary file 1 [file animals-12-02828-s001.zip › animals-1876060-supplementary.pdf]

Table S1. Breed pedigree information: maximum number of fully (FullGen), total (MaxGen), and equivalent complete (EquiGen) traced generations, % of subjects with a complete first generation and with MaxGen equal to 1, and effective population size (N_e).

Species/Breed	N. subjects	Maximum FullGen	Maximum MaxGen	Maximum EquiGen	Complete 1st generation (% subjects)	MaxGen = 1 (% subjects)	N_e
Camosciata delle Alpi	2093	6	21	8.8	54.3	8.8	1745
Saanen	935	7	23	10.4	55.0	8.5	2244
Sarda	1053	9	40	12.8	52.8	4.1	50087
Delle Langhe	104	9	33	13.2	75.9	1.2	456
Comisana	529	7	20	10.2	34.1	15.8	1229
Massese	371	8	27	13.3	59.9	9.2	1381
